# Feasibility of a clearing house for improved cooperation between telemedicine networks delivering humanitarian services: acceptability to network coordinators

**DOI:** 10.3402/gha.v5i0.18713

**Published:** 2012-10-09

**Authors:** Richard Wootton, Laurent Bonnardot, Antoine Geissbuhler, Kamal Jethwani, Carrie Kovarik, Suzanne McGoey, Donald A. Person, Anton Vladzymyrskyy, Maria Zolfo

**Affiliations:** 1 Norwegian Centre for Integrated Care and Telemedicine, University Hospital of North Norway, Tromsø, Norway; 2 Faculty of Health Sciences, University of Tromsø, Tromsø, Norway; 3 Department of Medical Ethics and Legal Medicine, Paris Descartes University, Paris, France; 4 Fondation Médecins Sans Frontières, Paris, France; 5 Department of Radiology and Medical Informatics, Geneva University, Geneva, Switzerland; 6 Center for Connected Health, Boston, MA, USA; 7 Harvard Medical School, Boston, MA, USA; 8 Department of Dermatology, University of Pennsylvania, Philadelphia, PA, USA; 9 Wayne State University School of Medicine, Detroit, MI, USA; 10 Pacific Island Health Care Project, Tripler Army Medical Center, Hawaii, USA; 11 Donetsk National Medical University, Donetsk, Ukraine; 12 Department of Clinical Sciences, Institute of Tropical Medicine, Antwerp, Belgium

**Keywords:** access, telemedicine, developing countries, network collaboration, humanitarian services

## Abstract

**Background:**

Telemedicine networks, which deliver humanitarian services, sometimes need to share expertise to find particular experts in other networks. It has been suggested that a mechanism for sharing expertise between networks (a ‘clearing house’) might be useful.

**Objective:**

To propose a mechanism for implementing the clearing house concept for sharing expertise, and to confirm its feasibility in terms of acceptability to the relevant networks.

**Design:**

We conducted a needs analysis among eight telemedicine networks delivering humanitarian services. A small proportion of consultations (5–10%) suggested that networks may experience difficulties in finding the right specialists from within their own resources. With the assistance of key stakeholders, many of whom were network coordinators, various methods of implementing a clearing house were considered. One simple solution is to establish a central database holding information about consultants who have agreed to provide help to other networks; this database could be made available to network coordinators who need a specialist when none was available in their own network.

**Results:**

The proposed solution was examined in a desktop simulation exercise, which confirmed its feasibility and probable value.

**Conclusion:**

This analysis informs full-scale implementation of a clearing house, and an associated examination of its costs and benefits.

Telemedicine, which can be defined as ‘medicine at a distance’, is practised in many forms. One form of telemedicine involves the delivery of services to assist doctors who work in developing countries. Here resources are often limited, and assistance with diagnosis and management may be difficult to obtain. Several networks exist for the purpose of supporting doctors in this situation, usually by providing specialist advice to queries about patients, or providing interpretations of imaging or pathology results. This is an example of the more general use of e-health systems to facilitate care at a distance (1).

A recent review identified seven telemedicine networks, each of which had been in operation for at least 5 years and which provided store-and-forward services to doctors in low- and middle-income countries. These networks provide clinically useful services and improved healthcare access (2, 3). In order to do so, the telemedicine networks maintain a panel of specialists to advise the referring doctors. In practice, there are occasionally instances when a particular expert is not available, and the networks then engage in an informal process to identify suitable specialists in other networks. This process is rather laborious and time-consuming. Recently, it has been suggested that a mechanism for sharing expertise between networks might be useful (4). To discover whether this would actually be the case, it would need to be implemented and tested. Before doing so, however, its feasibility needs to be established.

The aim of this study is to propose a mechanism for implementing the ‘clearing house’ concept for sharing expertise and to confirm that it would be feasible in the sense of being acceptable to the long-running telemedicine networks identified in the previous review. For the purposes of the present work, only dialogue between participants in a single language (English) is considered, since multilingual telemedicine, while perfectly possible, presents additional complexities.

## Setting

The seven networks studied in the previous review have been in operation for a median duration of 11 years (range 5–15) (2, 3). They all provide clinical teleconsultations for humanitarian purposes, and five of them are also involved in some form of education. Given its similar characteristics, the more recent English-speaking network of Médecins Sans Frontières (MSF, or Doctors Without Borders) was also included in the present study. The MSF telemedicine system was launched in 2009 and was based on the design used by the Swinfen Charitable Trust (5). The MSF system operates telemedicine networks in three languages (English, French, and Spanish), with about 260 expert consultants and 200 referring doctors.

All networks use store-and-forward (asynchronous) methods for delivering teleconsultations. In addition, one telemedicine network uses videoconferencing for consultations regarding trauma cases, which can require an immediate response. Four of the networks offer teleconsultations in all clinical specialties, whereas the other three networks focus on the delivery of specific services, including trauma, orthopaedics, neurosurgery, dermatology, and HIV/AIDS.

In those networks providing educational services for health professionals, tele-education is mainly delivered via asynchronous methods such as computer-based learning or web-based discussion forums. Synchronous tele-education delivered through videoconferencing is also used by two networks. Educational activities are mainly offered by the telemedicine networks delivering specialty-related teleconsultations. Consistent with the clinical activities, tele-education is offered for trauma, dermatology, and HIV/AIDS.

The review showed that the number of requesters in each network ranged from 10 to over 500 (2, 3). The number of requesters was loosely associated with the range of clinical services provided, but not with the duration of network operation. The number of referring sites ranged from 4 to 399, while the number of countries ranged from 1 to 58. The number of sites and countries where requesters were based was roughly proportional to the number of requesters.

The smallest network had a total of 15 experts and the largest had about 500 experts. These were located in a number of sites ranging from 1 to 502, while the number of countries ranged from 1 to 22. The different telemedicine networks used different organisational models. One network noted that not all the requesters and experts were active, due to continual staff turnover. This is to be expected, especially for big networks where hundreds of doctors are registered. All the telemedicine networks had experts based in other industrialised countries. In addition, three networks also had experts located in the same countries where the requesters were based, while two networks had experts from other developing countries. The experts mainly worked as volunteers for the telemedicine network. Only in two networks were the experts paid for the time spent in delivering teleconsultations.

The management of requests and the selection of the experts responsible for answering them were done by a coordinator in seven of the eight networks. Thus the coordinator appears to be a key element underpinning the organisational model of the telemedicine networks. In one network, this activity was done entirely by the requesters themselves, while in another network the requesters were supported by a coordinator in the process. Coordinators were volunteers in four of the eight telemedicine networks, while the remaining four networks paid for their time.

The telemedicine networks were operated by a range of organisations. One was a charity, several were healthcare providers (e.g. university hospital groups or a medical non-governmental organisation) and one was run by the military.

## The problem

The goal of humanitarian telemedicine networks is to provide healthcare staff in low-resource areas with access to specialist expertise. The specialist expertise may be provided in the form of responses to clinical queries, that is, information relating to a specific patient, or in the form of continuing education, that is, information of more general nature.

The success of the operation depends largely on volunteer participation. For example, each network maintains its own list of specialists who volunteer to answer consultation requests. Problems arise when a consultant is either not available due to their personal schedule or when a network does not have a particular type of specialist on its books. For example, there may not be a specialist with the specific competence required for an unusual case, or if there is, the specialist may not speak the necessary languages. When this occurs, there is often collaboration between networks – specifically, to identify an expert in another network – but this is laborious and time-consuming. Coordinators contact each other by email and attempt to identify an appropriate expert who agrees to help, but the process is slow. Therefore, an easy-to-use, rapid method would be desirable.

Note that the obvious method of a coordinator manually searching the databases of the other networks is not practicable, due to privacy and security constraints. For example, one telemedicine network is operated by the military. While this network comprises numerous specialists who might in principle assist other networks, it would not be possible to grant database access to non-military personnel. In the same way, strict confidentiality with restricted access is a condition for many humanitarian organisations to allow them to work in developing countries.

How big a problem is this in practice? We conducted a needs survey of eight humanitarian networks for a period of 28 days, during which each coordinator recorded any occasions on which they had difficulty in identifying an appropriate expert from within their own network. During the study period, a total of 212 (clinical) cases were managed by the networks. There were eight cases (4%) in which a clearing house might have been used to identify out-of-network experts (Table 1).

**Table 1:** Results of the needs survey over a 28-day period (clinical cases only, educational work not considered)

	Type*	Cases	Cases where a clearing house might have been useful
Africa Teledermatology Project	S	21	0
ITM Telemedicine	S	5	0
MSF (English-speaking network)	G	17	1
Pacific Island Health Care Project	G	17	0
Partners Online Specialty Consultations	G	18	0
RAFT	G	83	1
Swinfen Charitable Trust	G	43	4
Teletrauma	S	8	2
*Total*		*212*	*8 (4%)*

* S=specialist cases, e.g. dermatology, HIV, or trauma; G=general cases.

Three of the telemedicine networks provide telemedicine services in a single specialty area (dermatology, HIV, or trauma), rather than in all specialties. While it is less likely that these specialist telemedicine networks would require an external expert, those networks might be very valuable in supplying experts to other networks. That is, the clearing house would be strengthened by the participation of all types of networks, both specialist and general.

## Aim

The goal of a clearing house is to improve the communication between humanitarian telemedicine networks with the object of providing improved access to specialist services, both for clinical and educational purposes (4). To do this, there must be a mechanism for each network to request aid from out of network specialists, while not interfering with the privacy and security requirements of other networks. This implies that each participating network would identify from its own pool of experts those who would be willing to assist other networks. It also implies that participating networks would have a mechanism for making this information available to the clearing house and keeping it up to date.

## Methodology

To propose a mechanism for implementing the clearing house and confirming its feasibility, we sought the willingness of key stakeholders to participate; most were coordinators of humanitarian telemedicine networks. Collectively, we developed a range of possible solutions. The feasibility of these solutions was then considered. Our final proposal integrates the initial solution with additional ideas that were identified during this research process. The proposed solution was run in pilot form between two networks over a 1-year period. After the pilot trial, the proposed solution was finalised and then tested in a desktop simulation by all the networks (often called a table-top exercise).

## Possible solutions

There are several mechanisms by which a coordinator could use the clearing house to identify an out-of-network expert. Most simply, a combined database could be stored in a clearing house server and accessed via a secure web browser. The coordinator in need could then conduct a manual search of this database. An alternative would be a technical interface in which the data in the central clearing house were integrated into the data in the coordinator's own database. In this case, if an in-network consultant was not available, the data in the central database could be accessed automatically without the coordinator needing to interact with the clearing house directly. While the latter option would require each network to modify its software, ultimately the practice of identifying an out-of-network consultant would be less time-consuming.

Following discussions amongst the key stakeholders, four possibilities emerged:

Option 1. establish a mechanism for coordinators to search directly in each other's network databases;
Option 2. establish a mechanism for a query to be launched that would search automatically in the network databases (e.g. analogous to the Internet WHOIS query);
Option 3. copy the relevant data to a centrally accessible database for the coordinators to search there; and
Option 4. integrate the data from the foreign networks into each coordinator's own database.

In all cases, it is assumed that the specialists in each participating network would have self-identified themselves as willing to assist networks other than their own.

The advantages and disadvantages of the four scenarios are summarised in Table 2. After considering these possibilities, the following solution was proposed.

**Table 2:** Four possible methods of enabling coordinators to access information in other networks

Option	Mechanism	Pros	Cons
1	Coordinator can log into and search in the databases of all the other networks	(a) No modification of existing network software required, e.g. each coordinator would have user accounts on the other networks	(a) Coordinator would need to learn multiple user interfaces (b) Networks with special security requirements (e.g. those operated by the military, or by certain aid agencies that work in conflict zones) would need to agree to access by personnel from outside their organisation
			(3) Slow, since the coordinator would need to access and search each network one at a time
2	Coordinator can launch an automatic search query while remaining logged into own network (i.e. search takes place in all the network databases)	(a) Fast (b) Coordinator could launch query from within own network and would not need to learn new user interfaces	(a) Relatively complex software modifications required in all participating networks
3	Coordinator can search in a central database maintained by the Clearing House	(a) Fast (b) Coordinator does not need to learn user interfaces of all other participating networks	(a) Some software modifications required in all participating networks (b) Some IT resources required to establish and maintain the Clearing House (c) Requires coordinator to log into Clearing House system
4	All network databases are integrated, so that coordinator can search while logged into own network (i.e. search takes place within the coordinator's database)	(a) Fast (b) Does not require additional search performed by coordinator	(a) Relatively complex software modifications required in all participating networks (b) Networks must agree to access by out of network personnel

## Proposal

The consensus amongst the stakeholders was that Option 3 represented a simple approach that could be trialled relatively easily. That is, the clearing house would hold a central database containing information about consultants who had agreed to provide help to other networks. This database would be made available to network coordinators who needed a specialist when none was available in their own network. In this scenario, only limited data would need to be stored centrally:

1. type of specialist (note that an agreed taxonomy might be required for efficient searching);
2. identity of the network;
3. date of the last telemedicine case handled (this information is useful to a coordinator in deciding whether to use the specialist);
4. total number of telemedicine cases handled (also useful in deciding whether to use the specialist); and
5. recency of the information, that is, the date of last update by the host network.

**Fig. 1:**
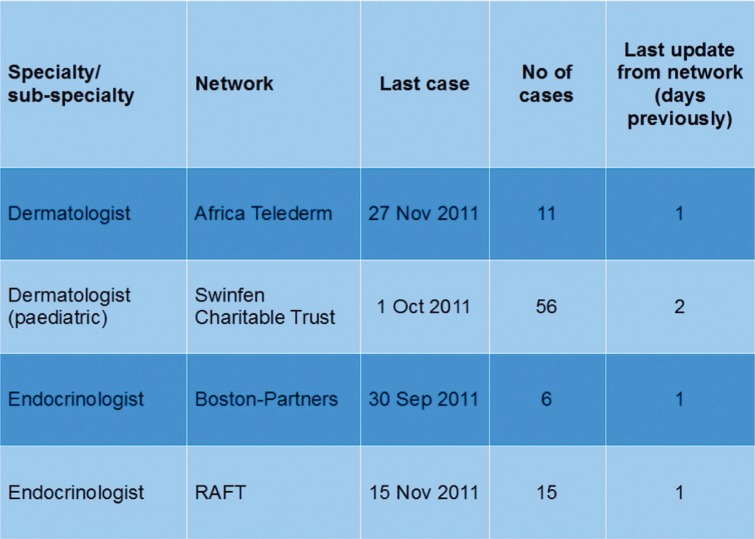
Basic information that might be made available in a clearing house.

This basic information is anonymous, and centralised storage does not raise significant privacy and security concerns. The details could be elaborated by including a brief profile of each specialist, perhaps containing additional information about their particular professional expertise, experience in developing countries, languages spoken, and so on. However, there is a trade-off between the desire for maximum information and the objective of storing simple and anonymous data. Nonetheless, it might be useful to supplement the basic information with the specialist's email address to speed up the process of making contact. An example screen is shown in Fig. 1.

Once an appropriate consultant was identified in the central database, the coordinator in need would be put in touch with the chosen specialist. There are two methods by which the specialist could be contacted. One possibility is that the names and email addresses of volunteer participants would be held in the clearing house database, allowing the coordinator in need to contact them directly. A copy of the contact email could be sent to the coordinator of the hosting network so that they would remain aware of the activities of their consultants.

The other method would be to store the specialist information in anonymised form, but with the identifying code that is used in their own network. In this scenario, the coordinator in need would have to contact the host network coordinator in order to obtain the specialist's email address. This places a higher demand on the second coordinator. There are ways to streamline this process, such as sending automatic emails from the database, but the host coordinator would ultimately be responsible for providing the contact information for their specialist to the coordinator in need.

**Fig. 2:**
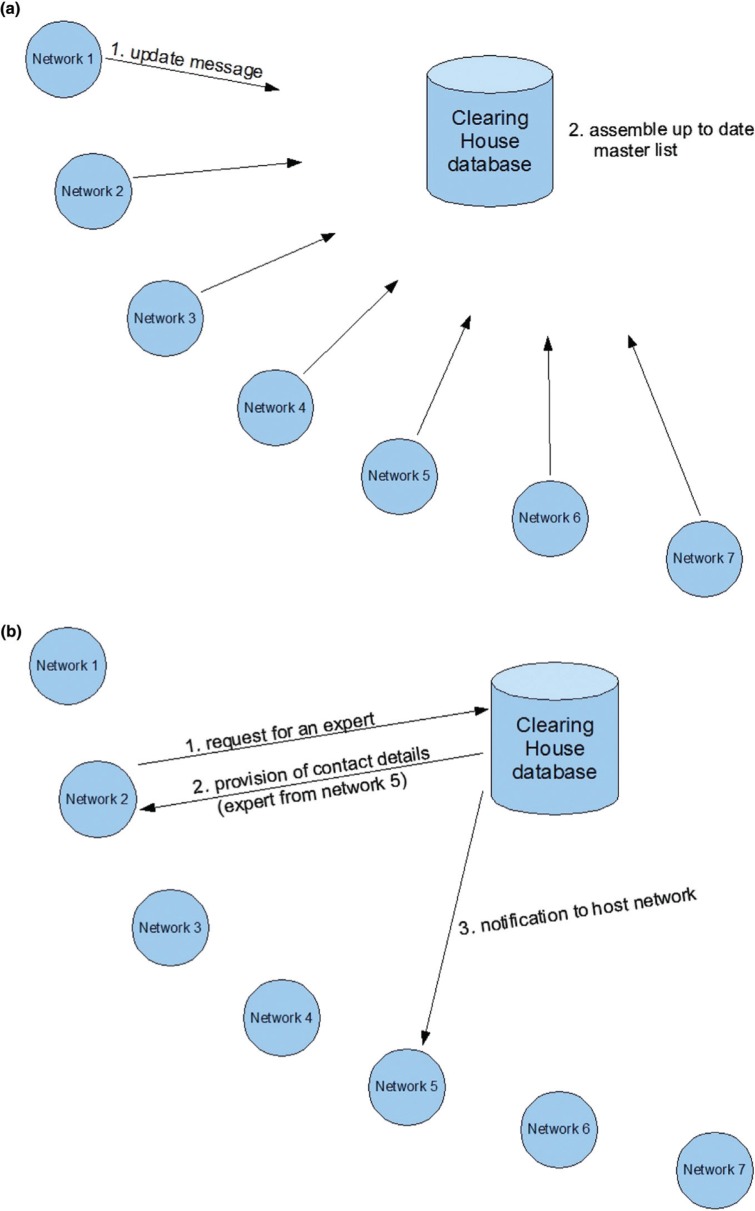
Clearing house search process. (a) A list of volunteer specialists is assembled (probably once a day); (b) the coordinator in need searches for a particular expert.

We propose using the first method which is significantly less burdensome for the second coordinator. With an automated copy of email communications, they can be kept aware of the activity of their consultant without being responsible for contacting them (Fig. 2).

In both methods, email would be the point of initial contact with the out of network expert, but it would not be the means used to send clinical information. The coordinator in need would have to provide the chosen specialist with login details and information on how to answer the consultation using their network's system. This is a potential disadvantage, because the chosen specialist will need to log in to an unfamiliar system in order to respond to the consultation. On the other hand, the specialists who do this will have specifically volunteered beforehand to assist networks other than their own.

## Updating the clearing house

For successful operation, the clearing house will need to maintain an up to date list of consultants who have volunteered to assist networks other than their own. While an initial list of consultants could be assembled manually, it would need to be kept up to date, because of staff turnover, changes of email address, and so on. Keeping it up to date would be very labour-intensive, so it would be better if the central database was updated automatically by a program that assembled a list of volunteer consultants. This list would be assembled from reports submitted by the participating networks. Thus, each network would run a program automatically, probably once a day, to scan its own list of available consultants and send an update message to the clearing house. The clearing house in turn would need to receive these reports, integrate them, and promulgate them in a master list.

Note that the clearing house would also need to identify duplicate records in cases where a particular consultant belonged to more than one network.

## Confirmation of feasibility: small-scale pilot

During a 12-month period beginning in July 2011, an informal clearing house was operated between coordinators of two of the participating networks to prove the concept. During the study period, one network managed a total of 247 cases and the other managed 500. Difficulties arose in identifying suitable consultants on a total of 67 occasions (20 and 47 cases, respectively), that is, in 9% of the cases managed by the two networks. ‘Difficulty’ was defined here as a failure to obtain a specialist response on three attempts or a delay in receiving a response of greater than 48 hours. These difficulties were the result of non-availability of the required specialist, perhaps because of a lack of the requisite language skills or because the request was relatively urgent and required a rapid response. On six occasions (two in the first network and four in the second), consultants from the other network agreed to provide assistance. Subsequently, these consultants became members of two networks rather than one.

## Confirmation of feasibility: desktop simulation

**Fig. 3:**
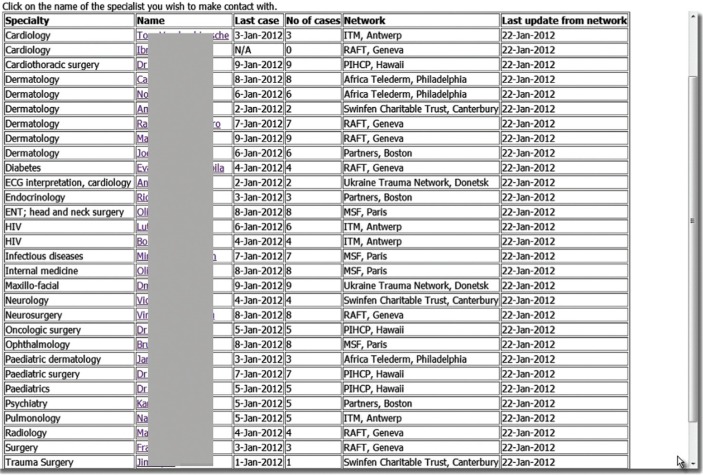
Simulation of proposed clearing house.

The network coordinators who were contacted for the present study agreed that Option 3 above (see the section on Possible Solutions) represented a viable approach. This model involves the relevant data being stored in the clearing house database and accessed by the coordinator in need. To test the feasibility of this model, each coordinator was asked to provide the names of 2–3 consultants in their networks who would be likely to volunteer to answer out-of-network queries if invited to do so in the future. These details were then collated manually to produce a web page that was made available in a simulation of the clearing house (Fig. 3).

Login details were provided to each network coordinator, and they were asked to imagine that queries received over the next 4 weeks could not be answered because of a lack of a suitable consultant in their own network. The web page was consulted and if an appropriate expert was listed, the coordinator could click a hyperlink which generated an email message for them. This message, which was copied to the coordinator at the host network for information, contained further details about the chosen specialist, including their email address (not shown on the web page). The coordinator could then contact the specialist directly and provide the necessary details to enable them to respond to the consultation.

During this desktop simulation, a number of (relatively minor) suggestions were made about improving the automatic notification messages.

## Discussion

Ensuring equitable access to healthcare is a well-known problem, even in industrialised countries, but a particularly difficult one in low- and middle-income countries. Telemedicine is often proposed as a method of reducing inequities, but there is little experience on an international scale. High bandwidth, satellite links are sometimes proposed for international telemedicine, but in practice useful telemedicine can be performed using low bandwidth, store-and-forward techniques, such as email and web-messaging (2, 3). Indeed, so-called delay tolerant networking appears to be an attractive potential method of obtaining network connectivity in low-resource settings (6).

The present study confirms the likely value of improved mechanisms for cooperation and sharing of information between telemedicine networks delivering humanitarian services. Although difficulties in finding a specialist within a given network occur in a fairly small proportion of cases – say 5–10% – methods of improving the chance of obtaining a specialist response are very valuable to those responsible for network operations and will boost the confidence of referring doctors that timely and appropriate second opinions can be obtained as a matter of routine.

With the assistance of key stakeholders, a number of options were examined, and a simple method was proposed. A central database would satisfy the needs of coordinators to identify out of network experts without the laborious task of contacting each network individually and then obtaining the expert's approval before sending them the case. The concept was tested in a small-scale pilot. A central database was then simulated and trialled successfully in a table-top exercise. This demonstrated its feasibility.

Real-world implementation depends on identifying a suitable host for the clearing house, and the willingness of participating networks to identify volunteer specialists and keep the information up to date. It is to be hoped that the advantages of belonging to the clearing house will enable the necessary resources to be provided within each network.

Although the present study trialled a simple solution to the problem of sharing information between participants with strict privacy and security requirements, more sophisticated approaches are possible, and may prove desirable in the longer term. For instance, rather than dealing with a centralised database, the system could evolve towards a federated registry which could be queried by software tools, for example, through a web-service interface. Such an implementation of a ‘network of networks’ would follow current distributed directory models or recent e-health standards dealing with ‘cross-institutional document exchanges’ that provide distributed maintenance of lists and decentralised access control. This type of system may become easier to implement as healthcare organisations move towards greater incorporation of information technology into their daily activities.

In the present work we have focused our attentions on single-language networks, in the interests of simplicity. However, one of the potential benefits of the clearing house is to overcome language boundaries by providing access to specialists who can work in different languages. For this reason, the limited data shown in Fig. 1 might be supplemented by information about specialists who can provide advice in more than one language.

Finally, it would be important to establish guidelines for use similar to those previously suggested (4). Ultimately, the goal would be to improve communication between networks thus allowing humanitarian networks to answer all requests even when an in-network consultant was not immediately available.

## Conclusion

There is a growing worldwide trend towards the use of IT devices, smartphones, the Internet, and social networks. In low- and middle-income countries, such technologies are increasingly employed for health purposes, both by healthcare staff and by patients and members of the public (7). A recent survey identified 176 technology-enabled programmes, of which 17% were designed to improve diagnosis and treatment, usually involving communication with a health worker (8). Clearly, there is an increasing need for extending telemedicine services and, in particular, for obtaining specialist medical advice. A clearing house might significantly facilitate and improve the efficiency of communication between telemedicine networks that share similar approaches and objectives. The present study confirms the feasibility and probable value of improved mechanisms for cooperation and sharing of information between telemedicine networks delivering humanitarian services. The next step is the full-scale implementation of a clearing house and an examination of its costs and benefits.
